# Venture Capital's Role in Advancing Plastic Surgery

**DOI:** 10.1093/asjof/ojae064

**Published:** 2024-08-27

**Authors:** Ravi Dhawan, Denys Shay, Kendall Brooks, Albert Losken

## Abstract

Plastic surgery sector has experienced significant growth, driven by an aging population's demand for minimally invasive procedures and technological innovation. Despite this, the role of venture capital (VC) in driving innovation within this sector remains underexplored. This study aimed to analyze the trends in VC investments in plastic surgery over the last 20 years, providing insights into the financial landscape of the sector. A retrospective cross-sectional analysis was conducted on VC investments in plastic and aesthetic surgery companies worldwide from January 1, 2003 to December 31, 2023, utilizing PitchBook (Seattle, WA). Companies were categorized into therapeutic and thematic areas, with investment trends analyzed by deal and company values, average investment size, and total capital invested. The study found 543 VC firms made 402 investments in 163 companies, totaling $1.98 billion, increasing by 15,143% over the study period, and focused on general plastic surgery and facial cosmetic procedures. Significant investments were also made in surgical software (25.3%), biotechnology and drug discovery (22.8%), and clinic and outpatient services (20.3%). Geographically, most investments were made in companies registered in Asia (45%) and North America (33.2%). VC investments have contributed to the growth and innovation in the plastic surgery sector, particularly in minimally invasive devices. The focus on general and facial cosmetic surgery aligns with market demand trends for aesthetic procedures. These findings underscore the importance of VC in shaping the future of plastic surgery and suggest the potential for strategic investments to further drive innovation.

**Level of Evidence:5 (Diagnostic):**

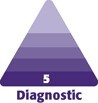

The plastic surgery sector is a significant part of the global healthcare market, with over 26.2 million procedures conducted annually and contributing billions of dollars to healthcare expenditures. The sector's growth is primarily driven by an aging population and increased demand for minimally invasive cosmetic procedures.^1,2^ Between 2019 and 2022 alone, aesthetic procedures experienced a 37% increase, accounting for $11.8 billion in annual revenue.^3^ Alongside market growth, the field of plastic surgery has seen considerable technological innovation. Between 1960 and 2010, the field was awarded >4600 patents for a range of innovations, including novel injectables, reconstructive prostheses, implants, and advancements in tissue engineering.^4^ These technological advancements have expanded the capabilities of plastic surgery practice and significantly improved patient outcomes.^4,5^ However, although capital infusion from venture capital (VC) is known to be fundamental in driving innovation in healthcare, it remains understudied in the context of plastic and aesthetic surgery.^6-10^ This study aims to address this gap by analyzing the trends in VC investments in plastic surgery over the last 20 years, and may help surgeons and entrepreneurs understand the financial landscape of surgical care, facilitating a deeper comprehension of the sector's financial developments and supporting informed decision-making for future innovations. To our knowledge, this study represents the first 20 year analysis of VC investments in plastic surgery.

## METHODS

We conducted a retrospective cross-sectional analysis examining the acquisition and funding of privately held plastic and aesthetic surgery companies by VC firms from January 1, 2003, to December 31, 2023. We used PitchBook (Seattle, WA), a robust capital market database used extensively in previous research, to determine relevant companies, using search criteria specific to plastic and aesthetic surgery: (aesthetic surgery OR aesthetic treatment OR aesthetics services OR medical aesthetic services OR plastic surgery OR plastic surgeon OR botox OR cosmetic treatment OR breast reconstruction OR aesthetic skincare OR cosmetic surgery OR cosmetic enhancement OR medical aesthetic OR breast surgery OR breast implantation OR brow lift OR body contouring services OR facial plastic surgery services OR plastic surgery procedures OR hair transplant) AND (healthcare OR surgery OR healthcare Technology Systems OR medicine OR healthTech).^11,12^ Our search criteria excluded all dissolved and/or bankrupt businesses. These data were collected on January 25, 2024, and analysis of the data was completed between January 31, 2024 and February 15, 2024. We employed methods and data sources that have been used in previous analyses of private capital investments in healthcare companies.^8-10,13,14^

Two reviewers (R.D. and D.S.) independently categorized the 163 companies that were eligible for analysis, using information from publicly accessible company websites, into therapeutic categories (facial cosmetic surgery, breast cosmetic surgery, body contouring, breast reconstructive surgery, hair restoration, and general plastics). These therapeutic categories were tailored based on the most common surgical and nonsurgical procedures in plastic surgery, based on procedural demand, in the calendar years 2022 to 2023.^3^ We further categorized these companies into thematic categories relevant to plastic surgery (clinics and outpatient services, surgical software, biotechnology and drug discovery, minimally invasive devices, injectables, implants, and lasers). If a company was involved in multiple therapeutic or thematic categories, only their most applicable category was noted. In case of disagreements between reviewers a consensus was made after deliberation at the time of review between the reviewers.

We also conducted a detailed geographical analysis to identify the specific countries providing VC, particularly within broad regions such as Asia, Europe, and North America, allowing us to pinpoint the most active countries in funding plastic and aesthetic surgery companies. We identified the top-funded companies in plastic surgery, and provided examples and a characterization of those receiving the largest amounts of VC. Additionally, we detailed specific products and services that received funding. Finally, we characterized the VC firms or individual investors providing the funding to these companies.

## RESULTS

**Figure 1.**
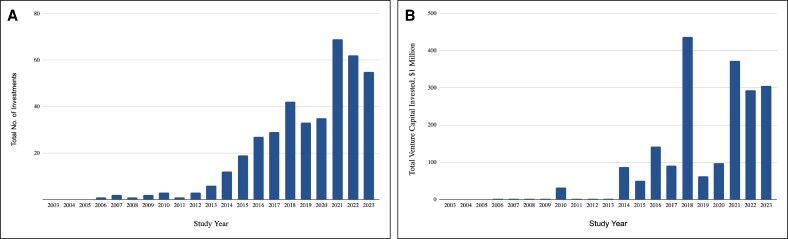
Annual VC investment in aesthetic and plastic surgery companies. (A) The annual number of VC investments in aesthetic and plastic surgery companies from 2003 to 2023. The *y*-axis represents the total number of investments, and the *x*-axis represents the study year. The data demonstrate a noticeable increase in the number of investments over the years, with a significant rise starting around 2013, peaking in 2021, and maintaining high levels through 2023. (B) The total amount of VC invested in aesthetic and plastic surgery companies each year from 2003 to 2023. The *y*-axis represents the total VC invested in millions of dollars, and the *x*-axis represents the study year. The investment amount saw substantial growth starting from 2014, reaching its highest point in 2018, with significant investments continuing through 2023. VC, venture capital.

**Figure 2.**
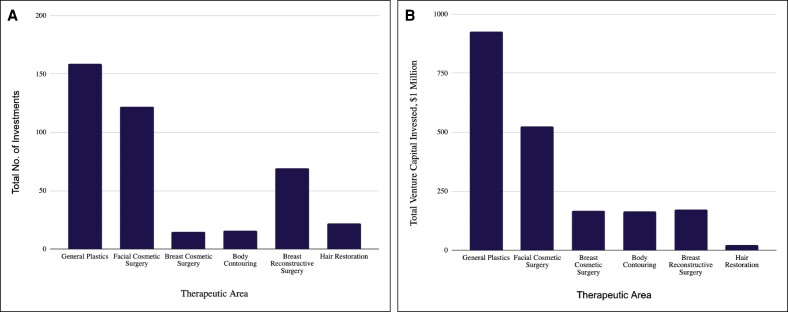
VC investment in plastic and aesthetic surgery companies by therapeutic area. (A) The total number of VC investments in different therapeutic areas of plastic and aesthetic surgery. The *y*-axis represents the total number of investments, and the *x*-axis represents the therapeutic areas. The data indicate that the highest number of investments was made in general plastics, followed by facial cosmetic surgery and breast reconstructive surgery with 159, 122, and 69 investments, respectively. (B) The total amount of VC invested in various therapeutic areas of plastic and aesthetic surgery. The *y*-axis indicates the total VC invested in millions of dollars, and the *x*-axis represents the therapeutic areas. The highest total investment is observed in general plastics ($924.0), followed by facial cosmetic surgery ($523.0) and breast reconstructive surgery ($166.9). VC, venture capital.

**Figure 3.**
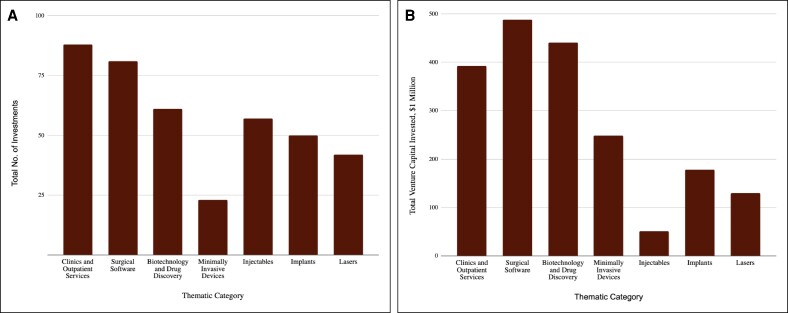
VC investment in plastic and aesthetic surgery companies by thematic category. (A) The total number of VC investments categorized by thematic areas within plastic and aesthetic surgery. The *y*-axis represents the total number of investments, and the *x*-axis represents the thematic categories. The data show that the highest number of investments was made in clinics and outpatient services, followed by surgical software and biotechnology and drug discovery with 88, 81, and 61 investments, respectively. (B) The total amount of VC invested in various thematic categories within plastic and aesthetic surgery. The *y*-axis represents the total VC invested in millions of dollars, and the *x*-axis represents the thematic categories. The highest total investment was observed in surgical software ($488.0), followed by biotechnology and drug discovery ($440.5) and clinics and outpatient services ($392.3). VC, venture capital.

From January 1, 2003 to December 31, 2023, 543 VC firms made 402 individual investments in 163 plastic and aesthetic surgery companies, totaling nearly $1.98 billion. The investments spanned from early companies to late-stage publicly traded companies with a 20 year average investment size of $7.09 million. From 2003 to 2023, the total annual capital invested in plastic surgery companies increased by 15,143% (Figure 1). Most investments (Figure 2) were targeted toward general plastic surgery ($924 million, 46.7%), and facial cosmetic procedures ($523 million, 26.5%). Breast cosmetic surgery ($166.9 million, 8.4%), breast reconstructive surgery ($173.8 million, 8.8%), and body contouring ($165.4 million, 8.4%), received similar amounts of investment, followed by hair restoration ($23.6 million, 1.2%). Furthermore, most investments targeted innovation (Figure 3) in surgical software ($487.9 million, 25.3%), biotechnology and drug discovery ($440.45, 22.8%), and clinic and outpatient services ($392 million, 20.3%). This was followed by interest in minimally invasive devices ($248.0 million, 12.9%), implants ($178.2 million, 9.25%), lasers ($129.6 million, 6.7%), and injectables ($50.9 million, 2.65%).

An analysis of companies receiving the greatest VC funding in plastic surgery revealed companies such as BeauCare Clinics (Beijing, China) in China, which received $118.7 million in VC funding for a range of cosmetic procedures, Ever/Body (New York, NY) in the United States which received $115.37 million for facial cosmetic treatments, as well as other notable companies (Table 1). Characterization of plastic surgery products receiving VC funding highlighted companies such as G/C Aesthetics (Dublin, Ireland), which raised $115.3 million for innovative breast implants, and Cytrellis (Woburn, MA), which raised $91.6 million for its microcoring technology to improve wrinkle appearance without scarring (Table 2). Furthermore, an analysis of VC investors in plastic surgery revealed top investors (Table 3), including Legend Capital, Beijing, China (5 investments), Montreux Equity Partners, San Francisco, CA (5 investments), and Redesign Health, New York, NY (5 investments).

**Table 1.** Characterization of Companies Receiving the Greatest VC Funding in Plastic Surgery

Company name	Description of company	Total capital raised ($1 million)	Company location	Therapeutic area	Thematic category
BeauCare Clinics	Operator of cosmetic surgery clinics offering plastic surgery, microbeauty surgery, minimally invasive procedures, and laser treatments	118.7	Beijing, China	General plastics	Clinics and outpatient services
Ever/Body	Provider of injectables and surgical facial cosmetic treatments aimed at wrinkle reduction and rejuvenation	115.37	New York, NY, USA	Facial cosmetic surgery	Clinics and outpatient services
GC Aesthetics	Developer of silicon breast implants technology to enhance female aesthetics, promoting a healthy, youthful, and confident appearance	115.0	Dublin, Ireland	Breast cosmetic surgery	Implants
Gengmei	Developer of a social networking app for planning and discussing cosmetic surgery, offering information, networking, and e-commerce for outpatient services	101.7	Beijing, China	General plastics	Clinics and outpatient services
Cytrellis	Developer of nonsurgical aesthetic technology, offering hand-held devices to eliminate sagging skin and improve age-related changes, enhancing patients’ quality of life	91.6	Woburn, MA, USA	General plastics	Minimally invasive devices
PatientFi	Operator of a service platform for healthcare expenses, specializing in plastic surgery and other specialties, to help patients access procedures	83.7	Irvine, CA, USA	General plastics	Clinics and outpatient services
Dexlevo	Developer of polymer technology for smart drug delivery in skincare and surgery, providing collagen stimulators and biodegradable polymers to enhance appearance	69.3	Seoul, South Korea	Facial cosmetic surgery	Biotechnology and drug discovery
RealSelf	Developer of a platform offering information and provider listings for plastic surgeries such as facelifts, liposuction, rhinoplasty, and hair transplants	58.4	Seattle, WA, USA	General plastics	Clinics and outpatient services
Trautec	Developer of recombinant collagen-based biomaterials for medical aesthetics, offering innovative human-derived collagen products for regenerative medicine	57.1	Jiangsu, China	General plastics	Biotechnology and drug discovery
Junhemeng	Developer of biological protein-based medications for medical aesthetics, focusing on recombinant A-type botulinum toxin and growth hormone for patient care	56.7	Hangzhou, China	General plastics	Biotechnology and drug discovery

VC, venture capital.

**Table 2.** Characterization of Plastic Surgery Products Receiving VC Funding

Company name	Primary products	Description of products	Total capital raised ($1 million)
G/C Aesthetics (Dublin, Ireland)	Specializes in innovative breast implants and tissue expanders for plastic surgery	Implants featuring ultra-cohesive, form-stable gels in textured, smooth, and microtextured surfaces. The implants incorporate gel technologies like BioQ and Emunomic	115.3
Cytrellis (Woburn, MA)	The primary product, Ellacor, is a microcoring technology designed to improve the appearance of wrinkles without scarring	Uses hollow needles to microscopically remove excess skin, unlike thermal-based methods. Typically, it requires three 30 min procedures with minimal pain and variable recovery times. FDA cleared for moderate-to-severe wrinkles. Features include a touchscreen interface and an ergonomic handpiece	91.6
Dexlevo (Seoul, Korea)	Specializes in a fully liquid PCL injectable, GOURI, to promote callogenesis for facial rejuvenation	GOURI, a PCL-based injectable, promotes collagen synthesis across the entire face, improving skin thickness, density, and elasticity without lumps. The solubilized PCL forms a durable 3D matrix for long-lasting effects, differentiating it from other injectables with a higher risk of complications in Phases I and II clinical trials	69.3
Dominion Aesthetic Technologies (Houston, TX)	EON, an FDA-cleared, noninvasive body contouring device	EON utilizes a 1064 nm laser to induce apoptosis in subcutaneous fat cells, with a touchless robotic arm providing personalized, noninvasive treatment. The jet-impingement cooling system ensures patient comfort, and clinical trials show significant fat reduction in a single session	33.2
Sofregen (Framingham, MA)	Biomedical silk protein products designed to facilitate soft-tissue regeneration, offering plastic surgeons natural biomaterials for durable and effective tissue support	Sofregen's silk protein platform utilizes fibroin for tissue support and regeneration. The developed products, such as SERI Surgical Scaffold, enhance collagen generation and tissue remodeling. In development are injectable fillers for facial and body aesthetics and advanced biological scaffolds for complex tissue support. These innovations leverage liquid and porous silk particles to mimic soft-tissue biomechanics.	22.9
AMiPHARM (Seongnam, Korea)	Injectable medications for localized fat reduction	The primary product, AYP-101, is an injectable composed of PPC, extracted from soybeans. PPC promotes fat reduction through apoptosis and lipolysis without causing adverse inflammatory responses. AYP-101 is under clinical development and aims to meet unmet needs in the cosmetic surgery market	20.5
Lattice Medical (Loos, France)	Specializes in bioresorbable implants for breast reconstruction postmastectomy and autologous hypodermis reconstruction	MATTISSE is a bioresorbable, 3D-printed tissue engineering chamber designed for personalized breast reconstruction after cancer, promoting natural tissue regeneration and resorption over time. The RODIN implant is a porous, 3D-printed structure for autologous hypodermis reconstruction, enhancing cell adhesion, proliferation, and angiogenesis, and gradually resorbing after achieving its purpose	16.0

3D, 3-dimensional; PCL, polycaprolactone; PPC, polyene phosphatidylcholine; VC, venture capital.

**Table 3.** Characterization of Top 10 VC Investors in Plastic Surgery

Company name	Company location	Type of investor	Total no. of investments in plastic surgery	Total no. of investments in all industries
Legend Capital	Beijing, China	VC	5	834
Montreux Equity Partners	San Francisco, CA	Growth/expansion	5	80
Redesign Health	New York, NY	VC	5	58
ACME Capital	San Francisco, CA	VC	4	194
Ceyuan Ventures	Beijing, China	VC	4	205
Denmark’s Export and Investment Fund	Nordhan, Denmark	VC	4	502
HongShan	Beijing, China	VC	4	1813
Oyster Capital Partners	San Francisco, CA	VC	4	15
Tech Incubator Program for Startups	Seoul, South Korea	Accelerator/incubator	4	1.704
1517 Fund	Telluride, CO	VC	3	140

VC, venture capital.

**Figure 4.**
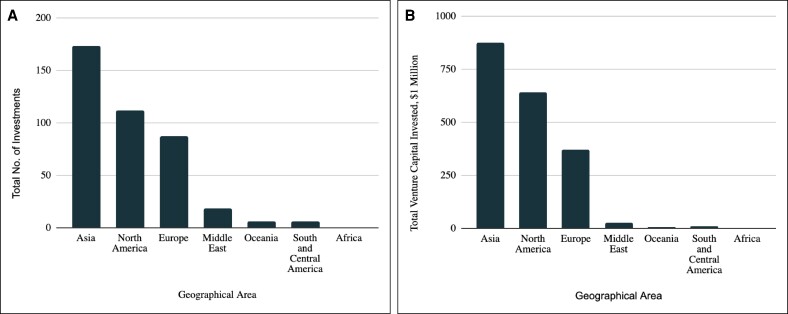
VC investment in plastic and aesthetic surgery companies by geographical area. (A) The total number of VC investments in plastic and aesthetic surgery companies across different geographical areas. The *y*-axis represents the total number of investments, and the *x*-axis represents the geographical areas. The data indicate that Asia received the highest number of investments, followed by North America and Europe with 173, 112, and 87 investments, respectively. (B) The total amount of VC invested in plastic and aesthetic surgery companies across different geographical areas. The *y*-axis represents the total VC invested in millions of dollars, and the *x*-axis represents the geographical areas. The highest total investment is observed in Asia ($874.1), followed by North America ($639.8) and Europe ($372.3). VC, venture capital.

The analysis of investment distribution revealed that the majority of investments were directed toward companies registered in Asia, which attracted $874.1 million (45%) of the total capital (Figure 4). North America followed with $639.8 million (33.2%), and Europe received $372 million (19.3%). Among the countries, China led with the highest number of companies (50, 28.2%) and deals (126, 31.3%), securing $770.3 million (38.9%) in total capital. The United States also showed significant investment activity with 40 companies (22.6%) and 109 deals (27.1%), totaling $618.5 million (31.2%). Ireland, despite having fewer companies and deals, received substantial investment (Table 4). South Korea, Denmark, France, Israel, Switzerland, Japan, and Italy also contributed to the overall investment landscape, each displaying unique patterns in deal count and capital allocation.

**Table 4.** Top 10 Countries Receiving the Greatest VC Funding for Plastic Surgery Companies

Country	Company count (%)	Deal count (%)	Total capital invested in millions (%)	Average capital invested in millions (%)
China	50 (28.2)	126 (31.3)	770.3 (38.9)	14.3
United States	40 (22.6)	109 (27.1)	618.5 (31.2)	6.7
South Korea	14 (7.9)	33 (8.2)	127.3 (6.4)	6.4
Ireland	2 (1.1)	7 (1.7)	218.8 (11.1)	31.5
Denmark	2 (1.1)	18 (4.5)	43.3 (2.2)	3.3
France	5 (2.8)	13 (3.2)	29.1 (1.5)	2.6
Israel	8 (4.5)	16 (4.0)	27.1 (1.4)	2.3
Switzerland	4 (2.3)	21 (5.2)	25.3 (1.3)	1.8
Japan	3 (1.7)	8 (2.0)	14.0 (0.7)	2.8
Italy	2 (1.1)	10 (2.5)	9.1 (0.5)	1.5
All other countries	33 (18.6)	41 (10.2)	97.2 (4.9)	3.0

VC, venture capital.

## DISCUSSION

In this cross-sectional study, we investigated VC investments in plastic and aesthetic surgery companies between 2003 and 2023. We found that 402 individual investments in 163 plastic and aesthetic surgery companies totaling nearly $1.98 billion were made, having risen by 15,143% from the start of this period. Most investments were targeted toward general plastic surgery and facial cosmetic procedures, with the greatest funding in surgical software and biotechnology companies registered in Asia and North America, in particular China and the United States.

Our findings show considerable investor interest in plastic and aesthetic surgery companies innovating or offering bundled services in general plastic surgery, and specifically, facial cosmetic surgery. With the growth of minimally invasive devices, predictive surgical software models, and surgical decision support tools, the potential for more efficient and effective procedures is evident. Accordingly, the 3 largest companies focused on the deployment of minimally invasive therapeutics and procedures for facial and breast cosmetic surgery. Several early-stage companies also focused on applications of artificial intelligence–based predictive models for cosmetic procedures. The focus of investor interest in companies offering services in general plastic surgery, facial cosmetic surgery, body contouring, and breast cosmetic surgery is in line with general demand: in 2022, the most common aesthetic procedures included liposuction, breast augmentation, and facial cosmetic procedures.^3^

Financial data corroborate the emerging narrative that the field of plastic surgery is increasingly gaining investor attention as a growth sector.^13,15,16^ Within the last decade, interest in the acquisition of plastic surgery practices by private equity groups has significantly increased: between 2019 and 2022 alone, strategic acquisitions of medical aesthetic companies by private equity corporations increased by 30% per annum, likely due to the growing profitability of minimally invasive and outpatient services.^17^ Moreover, the global plastic surgery market is expected to be valued at $59.5 billion by 2030, representing a compound annual growth rate of 3.4% between 2023 and 2030.^18^

The growth of investments is likely fueled by a wide variety of factors, ranging from changes in patient needs, attitudes, and behaviors to aesthetic procedures and innovations that are widening the accessibility of such procedures.^17^ The rise of social media use and trends on the likes of Instagram and TikTok have also accelerated the growth in demand for the sector.^19,20^

Plastic surgery entrepreneurs should be encouraged by the increasingly inefficient model of healthcare, in need of transformational discoveries and disruption. Each year, the US healthcare system loses between $760 and $935 billion due to inefficiencies, of which $286 billion could be saved through different forms of innovation.^21^ Aspiring plastic surgeons in academic settings who seek to address these inefficiencies through innovation have traditionally competed for selective NIH grants, which have become increasingly difficult to secure and are generally not designed for the commercialization of research.^22,23^

VC can be an alternative to traditional funding sources for surgeons committed to innovative projects that can both enhance patient outcomes and generate returns on investment for stakeholders. One model that has been proposed involves health systems as VC investors, who invest in surgeon innovators either within their institution or by cross-institutional collaboration.^22^ These models exist in some of the largest academic medical centers (AMCs) in the United States including Cleveland Clinic,^24^ Stanford University,^25^ and Northwell Ventures.^22,26^ So far, many of these investments driven by AMCs have been successful; research analyzing investments driven by this model between 2011 and 2019 found that <5% of the companies invested by AMCs had failed.^22,27^

However, many practicing plastic surgeons may not have access to VC funding through their institutions. Another method to bring early-stage ideas to the market involves partnerships with traditional venture capitalists, such as those described in this analysis, which can be developed through personal connections within the sector or networking at venues such as international plastic surgery conferences. After these initial connections are established, VC firms conduct thorough due diligence to ensure that the proposed surgical ideas align with VC firm's goals, advance medicine and plastic surgery and provide a return on investment.^28,29^

With the expansion in demand for aesthetics procedures, there is potential for strategic investors to merge smaller entities, such as medical practices, into larger networks. This strategy could enhance revenue for investors by offering a wider range of packaged services, including surgical, consumer cosmetic, and minimally invasive aesthetic solutions, tailored to changing consumer preferences. Innovating with subscription models for treatments, leveraging digital health to engage with patients,^30^ adjusting pricing strategies, streamlining surgical education through novel technologies,^31^ and improving insurance coverage^32^ are other ways for both innovators and investors to boost growth.^32^ Furthermore, increasing market saturation in developed countries may encourage both entrepreneurs and investors to innovate in developing economies such as India, where demand for cosmetic procedures is set to increase at an unprecedented pace in the next decade.^33^

Despite the potential for these developments, the involvement of VC firms in the financing of healthcare companies and surgeons involved in patient care is a topic of debate.^7,22^ While VC funding can provide essential capital for drug development and state-of-the-art aesthetic technologies, the primary goal of VC firms is to maximize enterprise value and ensure a substantial return on investment for shareholders. This may raise questions about the alignment of these financial objectives with optimal patient care.^6,7^ For instance, a surgical entrepreneur may face pressure from venture capitalists to optimize financial growth for their product and company rather than prioritize patient health.

Based on proposed AMC investment models, AMCs must thoroughly examine investments, ensuring that the objectives of their portfolio companies align with the priorities of the institution including, most importantly, patient well-being.^22^ Surgeon's innovating within AMCs and AMCs themselves should independently establish legal frameworks at the time of investment to address potential liabilities whether their companies engage in malpractice or inadvertently harm patients, leading to lawsuits.

This study is not without limitations. Our data source was limited to a single database, PitchBook, which does not include research on companies with nonpublic and undisclosed investments. Nevertheless, PitchBook is widely considered the most robust capital market database used by researchers and investors in several sectors.^11^ Our analysis, including 402 VC investments in 163 plastic and aesthetic surgery might also face underreporting issues due to nondisclosed transaction values. Misclassification bias could also be present since predetermined categories were used to group companies into specific plastic surgery subspecialties, possibly omitting some relevant investments or misclassifying companies. We aimed to mitigate this limitation preemptively by employing 2 independent reviewers. In addition, it was not possible to account for the factors that drove VC funds’ decisions to invest in plastic surgery technologies, or the proportion of successful or unsuccessful investments and companies in this field as these data were not completely available. PitchBook includes companies that have already raised VC and are inherently larger, and potentially less likely to fail, than smaller companies that do not disclose investments and therefore do not make it to the database. The purpose of our study was to examine relevant companies that have made or have the potential of making an impact in the field of plastic surgery. These topics could be the subject of future analysis should such data become available. Finally, the growth rates and investments calculated over the study period did not account for inflation, potentially skewing the results.

Despite these limitations, this study provides a novel analysis of VC investments in the field of plastic and aesthetic surgery. Our findings revealed the dynamic and growing landscape of VC investment in the plastic surgery sector over the past 20 years, particularly in innovative technologies like minimally invasive devices and artificial intelligence–driven procedures. The considerable investment in companies specializing in plastic and, specifically, facial aesthetic surgery aligns with increasing market demands for aesthetic procedures. Future research should focus on evaluating the impact of these investments on surgical advancements, compare VC funding to other financial mechanisms, and assess the broader implications for patient care and industry standards.

## CONCLUSIONS

VC investments have contributed to the growth and innovation in the plastic surgery sector, particularly in minimally invasive devices. The focus on general and facial cosmetic surgery aligns with market demand trends for aesthetic procedures. These findings underscore the importance of VC in shaping the future of plastic surgery and suggest the potential for strategic investments to further drive innovation.
